# Reconstruction of the Terminal of an Abandoned Fractured Unipolar Coronary Sinus Lead: a Feasible Solution to Restore Effective Cardiac Resynchronization Therapy

**DOI:** 10.1016/s0972-6292(16)30630-1

**Published:** 2013-06-25

**Authors:** Armando Gardini, Francesco Fracassi, Alberto Saporetti, Davide Mariggio

**Affiliations:** 1 Department of Cardiology, Istituto Clinico S. Rocco, Ome (Brescia, Italy); 2 Medtronic Italia, Sesto S. Giovanni (Milano, Italy)

**Keywords:** lead fracture, cardiac resynchronisation therapy, lead repair, venous obstruction

## Abstract

Complications related to coronary sinus lead are not infrequent in recipients of cardiac resynchronization devices. We describe the case of a patient with a biventricular implantable cardioverter defibrillator with persistent phrenic nerve stimulation, previous coronary sinus lead fracture, and severe left subclavian vein stenosis. The reimplantation of a new coronary sinus lead on the left side, ipsilateral to the original implant, was unsuccessful. In order to avoid more complex and risky procedures, we performed the repair of the fractured abandoned lead with the reconstruction of the unipolar lead terminal. Effective biventricular pacing was obtained with satisfactory electrical parameters and it was maintained at twelve months follow-up.

## Introduction

Resynchronization devices present a higher rate of complications than other implantable cardiac devices mainly related to the coronary sinus (CS) lead [1]. We describe the case of a patient with a biventricular implantable cardioverter defibrillator (ICD), subclavian vein stenosis, and intractable phrenic nerve stimulation (PNS) successfully treated with the repair of the terminal of an abandoned fractured CS unipolar lead. Possible alternative solutions are discussed.

## Case Report

**Figure 1:**
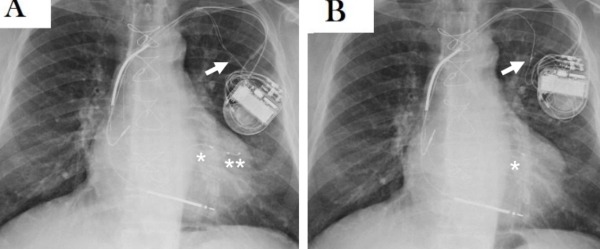
A) Chest PA X-ray showing the tip (*) of the unipolar CS lead fracture (arrow) near the tip of the bipolar CS lead (**); B) Chest PA X-ray showing the unipolar CS lead terminal repaired (arrow).

**Figure 2:**
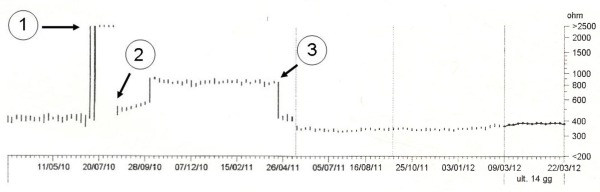
Trend of CS leads pacing impedance over time: 1) sudden increase corresponding to fracture of the unipolar lead; 2) reduction at time of reimplant of the bipolar lead; 3) return to original values at time of removal of the bipolar lead and repair of the fractured unipolar lead.

A 65-year-old man with history of myocardial infarction, previous coronary artery by-pass grafting, chronic heart failure with severe left ventricle (LV) dysfunction, and left bundle branch block, received a first biventricular ICD in 2003 with a dual coil defibrillation lead, a unipolar CS lead and a bipolar atrial lead. The device was replaced in 2005 due to a recall from the manufacturer and in 2009 for battery depletion. In August 2010 the patient presented haemodynamic decompensation, widening of the QRS and modification of paced-QRS axis. A check on the device showed an increase in the impedance and loss of capture of the unipolar CS lead. The chest X-ray revealed a fracture of the conductor of the CS lead in the subcutaneous pouch. A new bipolar CS lead was reimplanted via the left subclavian vein. During the implantation we tested different pacing configurations: the LV pacing thresholds were good and no PNS was observed with an output of 10 V. The fractured catheter was enveloped in thick and tenacious scar tissue, thus it was capped and abandoned in the pouch (Figure 1A). In February 2011 the patient started to complain of PNS which could not be corrected by changes in LV pacing configuration and output. A surgical revision was planned in order to check the catheter position. The bipolar lead was still inside a CS lateral side-branch. After insertion of a heavy-weight guide-wire which extended beyond the tip, we gently moved the catheter trying to restore a satisfactory position, without success. Then the bipolar CS lead was removed, leaving the guide-wire in the atrium via the subclavian vein. The insertion of an adequate size introducer for CS lead reimplant failed due to subocclusive vein stenosis despite the use of dilators with increasing diameter over the guide-wire. Repeated punctures of the vein downstream and upstream of the stenosis were also unsuccessful. Consequently we decided to restore the abandoned unipolar CS lead which was disengaged from the surrounding scar tissue. The insulation was transected proximally to the fracture point and pulled back. The conductor was stretched out and exposed beyond the insulation for a 0.5 cm portion. A 1.5 cm piece of a standard stylet was inserted into the lumen of the distal part of the lead so as to have a portion of a few millimetres extending beyond the conductor. The stylet was gently compressed with a clamp and secured with a silk tie and silicone glue in order to make the lead terminal stiff. Then, the fitted catheter was connected to a lead splice unipolar adaptor (VKU 17, Osypka AG, Germany) (Figure 1B). It was filled with silicone glue and secured to the body of the lead with a silk tie. The electrical parameters of the repaired electrode were good, comparable with previous chronic values. The pacing threshold was 1 V at 0.5 msec in the pre-fracture phase and 1.5 V at 0.5 msec in the post-repair phase. No PNS was observed at 10 V in the available configurations. The endocardial LV electrogram was good and no noise was recorded while moving and stretching the adaptor. At twelve months follow-up the patients was clinically stable and the repaired electrode showed adequate electrical parameters with unchanged pacing and impedance thresholds (Figure 2).

## Comments

Malfunction of the CS lead is the most frequent cause of surgical revision in cardiac resynchronization devices with a reported rate up to 8%. The most reported complications which require CS lead surgical revision are displacement, loss of capture, increased pacing thresholds, and PNS [2]. Stenosis of various degrees or occlusion of the subclavian vein has been reported in up to 25% of asymptomatic patients with chronic transvenous defibrillation leads. Severe stenosis (>75%) or complete occlusion occurs in about 15 % of cases especially in the presence of dual coil defibrillation leads and previous pacemaker leads [3]. Pacing lead fracture is a well known complication of cardiac pacing. The reported incidence varies widely depending on multiple factors such as lead design and materials, technique of implantation, and patient-related conditions like age and body habitus.

In our patient we had to solve two major issues, persistent PNS and severe venous obstruction, in order to restore effective biventricular pacing while considering the haemodynamic decompensation observed in concomitance with the loss of LV capture.

In case of obstruction of the subclavian vein ipsilateral to the initial implant site the replacement of a malfunctioning lead may be a problem. Different options should be attempted before considering complete controlateral reimplant. In fact this approach carries an increased risk for the development of superior vena cava syndrome and lead-lead interactions due to the increased number of leads. The feasible options proposed are endovascular implant via the controlateral subclavian vein with subcutaneous lead tunnelling, a variety of unconventional upper vein approaches, surgical epicardial implant, and subclavian vein venoplasty with or without lead extraction [4]. However all these approaches have several disadvantages and carry an increased procedural risk. We discussed with the patient the available alternatives and ultimately we agreed that the reconstruction of the terminal of the abandoned fractured CS lead was the more reasonable solution. It was a simple, feasible, and safe solution avoiding more complex and hazardous approaches. In fact epicardial implantation could result in failure or complications related to the presence of pericardial adhesions due to previous cardiac surgery. Moreover epicardial leads have a higher rate of malfunction and increase in pacing threshold than transvenous CS leads. Right side approach with subcutaneous lead tunnelling to the left sided pouch could be an alternative. However, in right-handed people it could cause right arm functional limitation and could expose the lead to lesion similarly to what occurred. Finally, extraction and reimplantation of the CS lead was considered but it was excluded because it could damage the atrial and right ventricular leads with the risk that complete extraction could become necessary.

No other case of CS lead repair with an adaptor has been reported to our knowledge. In the past before the mechanical or laser sheaths extraction era a similar technique was successfully used to repair damaged pacing leads that were chronically implanted [5]. More recently it was also proposed in pediatric patients with good results in order to extend the leads longevity and delay the time of the replacement [6].

According to our experience we can conclude that terminal lead reconstruction, if feasible, may be considered as a viable option also for LV pacing, as with other cardiac leads, in the presence of difficult venous access before attempting more complex and risky procedures.
